# Integrating ecological and community science data to understand patterns of colour polymorphism and social behaviour at the northern range limit of a plethodontid salamander

**DOI:** 10.1371/journal.pone.0332501

**Published:** 2025-09-23

**Authors:** Alexia McCormick, Julia L. Riley

**Affiliations:** Department of Biology, Mount Allison University, Sackville, New Brunswick; Laboratoire de Biologie du Développement de Villefranche-sur-Mer, FRANCE

## Abstract

Traditionally, scientists have relied on ecological surveys to gain information about wildlife; however, community-science data has recently emerged as a valuable resource in organismal research. In this study, we conducted ecological surveys in 23 forests in New Brunswick, Canada and extracted data from iNaturalist across the entire province to understand patterns of Eastern Red-backed Salamanders' colouration and sociality at the northern limit of its range. Ecological data revealed that adult salamanders were more likely to aggregate during the early spring and autumn, reflecting trends observed in other areas of their range. We also compared aggregation behaviour and colouration data between data collection methodologies and found that community-scientists are less likely to report aggregated salamanders and are more likely to report unique colour morphs than ecological surveys. Notably, iNaturalist observations included an amelanistic morph which had yet to be formally documented in New Brunswick. Lastly, we used our ecological survey data to explore if preferences for micro-environmental factors differ between colour polymorphism and aggregated vs. solitary salamanders. There was no evidence for environmental preferences in New Brunswick, which differs from tendencies observed in other populations of this species. Our findings highlight the trade-offs between ecological and community-science approaches and contribute valuable insights into the natural history of *P. cinereus* at the northern edge of its Canadian range.

## Introduction

Ecology has traditionally relied on field surveys to sample and learn about the natural history of organisms, but there are new emerging tools that can help researchers gain information about wildlife [1]. One such tool is iNaturalist, an online platform driven by community participation designed for recording and identifying observations of diverse taxa (http://www.inaturalist.org/). Through this platform, users upload an observation of an organism (with either a photograph or audio recording) using their phones or computers with or without a species’ identification and then receive suggestions from other community members, and/or iNaturalist’s built-in AI in newer software versions, both identifying the organism and verifying its identity. This generates new data on species occurrences, and each photo also includes other phenotypic (e.g., colouration), behavioural (e.g., con- and hetero-specific interactions), and conservation (e.g., threats to mortality, tracking species invasions) information [2,3]. Thus, community-science platforms, like iNaturalist, have become a valuable resource in wildlife research and conservation.

To date, researchers have used iNaturalist as a tool to study a wide breadth of traits across many taxa, including detailed accounts of variation in species colour and social interactions. Previous studies exploring such variables via iNaturalist include Surmacz (2023) who examined spatial patterns of a polymorphic flower species (*Convolvulus arvensis)*, and Farquhar et al. (2022) who explored patterns of geographic segregation among colour morphs of the Lace Monitor (*Varanus varius*) [4,5]. While less common, interactions between taxa have also been previously studied using iNaturalist images. For example, Bosenbecker et al. (2023) studied the interspecific interactions between plants and hummingbirds through a dataset generated through iNaturalist photographs [6]. Within this scope, the Eastern Red-backed Salamander (*Plethodon cinereus*) – a terrestrial salamander species found in woodland habitats in Eastern North America – emerges as a captivating subject for investigation through iNaturalist due to its distinct colour polymorphism and intricate social behaviour [7]. Both of these traits are likely shaped by environmental conditions, which makes this species particularly well-suited for examining how ecological factors influence social and phenotypic variation across populations.

The Eastern Red-backed Salamander (*Plethodon cinereus*) exhibits eight discrete colour phenotypes (albino, amelanistic, erythristic, iridistic, leucistic, melanistic, striped, and unstriped), four of which have been documented in New Brunswick, Canada (erythristic, leucistic, striped, and unstriped) [8]. Among these phenotypes, the striped and unstriped are the most common, and are considered distinct colour morphs – defined as genetically determined colour variants that occur within a single interbreeding population [9]. The striped and unstriped morphs are widespread throughout southeastern Canada and the eastern United States, although their proportions vary significantly between populations (Table S1 in S1 File) [10]. Several hypotheses have been proposed to explain the variation in morph frequency, however the most common explanation attributes it to contrasting environmental factors, with striped morphs more common in cooler, moister habitats and unstriped morphs more prevalent in warmer, drier environments [11–13]. Physiological differences between morphs – such as greater resistance to heat stress in unstriped individuals [14] – are consistent with this explanation and may reflect local adaptation to habitat conditions. Additionally, assortative mating by colour has been observed within populations [15], which may contribute to maintaining distinct morph frequencies over time. However, exceptions and contradictory findings have also been reported, with both morph frequencies and physiological traits varying considerably across populations, suggesting that these associations are not uniform across the species’ range [16]. Prevalence of the rarer colour morphs is similarly variable across the species’ range. For example the erythristic morph is sometimes considered rare, while in some populations is considered relatively common [17]. The remaining five phenotypes (iridistic, albino, leucistic, amelanistic, and melanistic) are much rarer anomalies with sporadic observations reported throughout this species’ range [8] – making their distribution less understood compared to the more prevalent striped and unstriped morphs.

In addition to colour polymorphism, environmental factors (e.g., seasonal fluctuations and microhabitat quality) also influence the social dynamics of *P. cinereus*, shaping how individuals interact and organize within their habitats. While this species is primarily territorial, it exhibits aggregative behavior under certain environmental circumstances, particularly during the cooler, wetter months (spring and fall) when individuals cohabit shared shelters in social groups of 2–7 [16,18]. This period of increased sociality coincides with the species’ mating season, during which mating pairs (i.e., two adults of opposite sexes within 30 cm of one another) are frequently observed [15]. The habitats these mating pairs are most likely to be found within (i.e., cool, moist microhabitats) are essential for females during egg-laying and parental care [19]. In contrast, during warmer, drier periods, solitary behavior is more typically observed as individuals spread out in response to increased intraspecific competition for limited resources [18]. The seasonal dynamics of these behaviors highlight the strong influence of environmental factors on the sociality of *P. cinereus*, yet much remains unknown about how these patterns play out in northern populations, such as those in New Brunswick, Canada [20].

Our goal was to increase understanding of two key biological characteristics of *P. cinereus* – colour polymorphism and social behavior – at its understudied northern Canadian range limit [21]. Community science platforms like iNaturalist present an exciting opportunity to investigate these two natural-history traits by providing access to a large dataset of *P. cinereus* observations, including over 10,000 observations of this species in Canada alone. Because of its widespread documentation, *P. cinereus* has already been the focus of studies examining such traits on iNaturalist; for example, Hantak et al. (2022) applied machine learning models to classify more than 20,000 color morphs of *P. cinereus* across its range from iNaturalist images [22]. While Naturalist records can be useful to document variation in such phenotypic and/or behavioural traits, the potential causal factors (e.g., macro- and micro-habitat, climate, genetics) driving this variation needs to be studied using an approach that allows collection of multiple, linked variables concurrently. Thus, we adopted a comparative framework to explore how field data and community-science observations each contribute to our understanding of *P. cinereus* colour polymorphism and social behavior at this species’ northern range limit. These two traits are examined together because they are both likely shaped by environmental conditions and have been shown in other contexts to be potentially interrelated – for instance, through assortative mating by colour morph [15]. Additionally, identifying the strengths and limitations of both community-science and traditional ecological approaches enables a more comprehensive understanding of *P. cinereus* phenotypic and behavioral variation, and may be able to guide future study designs in other under-studied regions.

We specifically tested whether data source (i.e., ecological field data versus iNaturalist data) influences our understanding of *P. cinereus* colour polymorphism and social behaviour in New Brunswick. We predicted that while the common striped morph would be similarly represented in both datasets, rarer morphs would be overrepresented in iNaturalist data, consistent with trends observed in other taxa [23]. Since iNaturalist has not been widely used to study intraspecific interactions, such as aggregations within species, we did not have specific predictions regarding how the source of data may influence our understanding of *P. cinereus* social behavior. Finally, we assessed whether environmental factors differed between observations of striped versus unstriped morphs, as well as solitary versus mating pairs/aggregations of salamanders. We hypothesized that striped individuals would be observed more often in cooler, moister microhabitats [11,12], and that mating pairs/aggregations would occupy higher-quality habitats (i.e., those with larger cover objects, higher moisture, lower temperature, and greater canopy coverage) compared to solitary individuals [24] because these habitats provide the conditions required for successful egg-laying and parental care [19].

## Results

**Fig 1:**
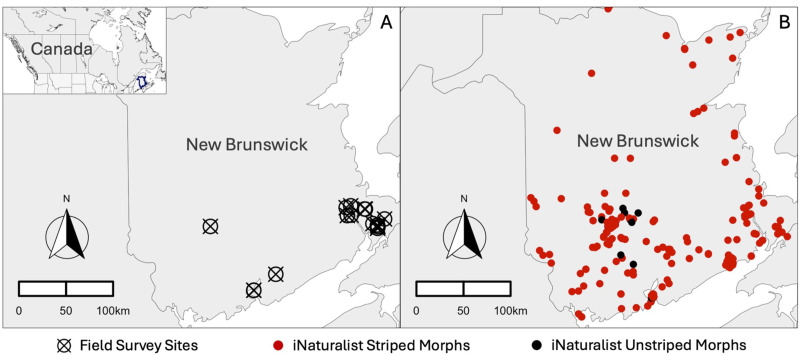
Location of field surveys (A), and iNaturalist observations (B) of Eastern Red-backed Salamanders (*Plethodon cinereus*) in New Brunswick. Each hollow circle with a cross represents the location of a field survey in New Brunswick (*n* = 23), each red circle represents iNaturalist reports of a striped colour morph (*n* = 263), and each black circle represents iNaturalist reports of an unstriped colour morph (*n* = 21). A small inset map in the top-left corner highlights the study area, New Brunswick, Canada, using a dark blue outline. The scale bar represents 100 km. The basemap was created using the ‘*ne_states’* function from the R package ‘*rnaturalearth’*, and plotted using the “*geom_sf”* function from the R package ‘*ggplot2*’ [25]. The scale bar and north arrow were added to plots using the *‘ggsn’* R package [26].

During a 6-month field study, we found 243 Eastern Red-backed Salamanders (163 adults, 73 juveniles, and 7 neonates) across 23 forests in New Brunswick (Fig 1, Table S2 in S1 File). Across all surveys, a total of 3,345 cover objects were flipped to search for salamanders. In each survey there was an average ± standard deviation of 111 ± 75 cover objects encountered. Yet, only about 6% of cover objects (195/3,345) had *P. cinereus* underneath*.* These inhabited cover objects (*n* = 195) ranged from 3 cm x 3.5 cm x 3.5 cm (length x width x height) to 32 cm x 223 cm x 770 cm, and were on average 172 cm x 17 cm x 12 cm. This is a surface area of 2,924 cm^2^. The cover objects *P. cinereus* used were 18% rocks and 82% logs.

### Colour morph summary and comparison

**Fig 2:**
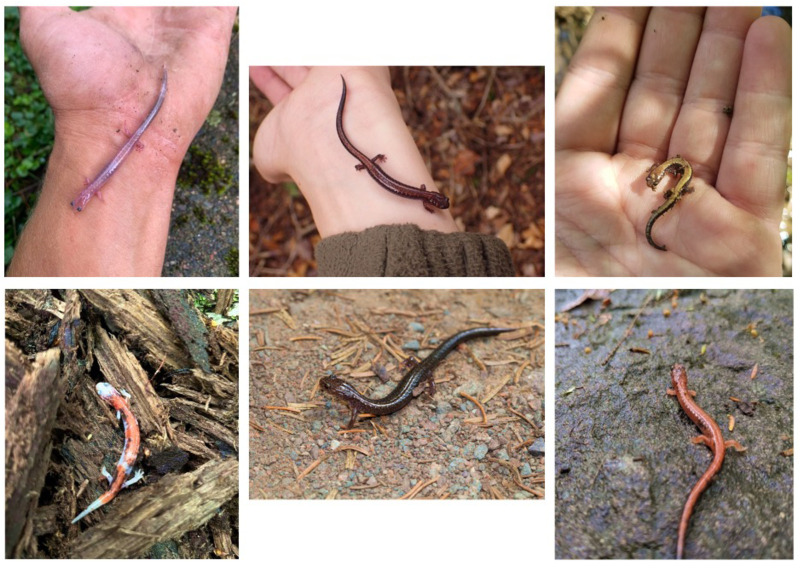
The colour variation in the Eastern Red-backed Salamander (*Plethodon cinereus*) in New Brunswick. All photos were reported on iNaturalist (http://www.inaturalist.org/) and included in this study. The top left individual is a leucistic morph (photo credit: Bradley Doiron), the top middle (photo credit: Alexis Godin) and top right individuals (photo credit: Sébastien Benoit) are red-backed or striped morphs, the bottom left individual is an amelanistic morph (photo credit: Damien Mullin), the bottom middle individual is a lead-backed or unstriped morph (photo credit: David Robichaud), and the bottom-right salamander is an erythristic morph (photo credit: Dani Landry).

Of the 243 *P. cinereus* found during our field surveys, a total of 233 salamanders were striped morphs, 8 were unstriped morphs, and 2 were erythristic (i.e., categorised as “other” in analyses). We downloaded data from observations of Eastern Red-backed Salamanders on iNaturalist from 2008 to 2022 from across New Brunswick, Canada. The iNaturalist dataset contained 316 research grade observations of the Eastern Red-backed Salamander, which consisted of 263 observations of the striped morph, 21 observations of the unstriped morph, and 15 observations of other morphs (i.e., categorised as “other” in analyses) (Fig 1; Fig 2). The other morphs that were reported included 12 erythristic salamanders, 1 amelanistic salamander, and 2 leucistic salamanders. The remaining 17 observations were unidentifiable due to issues with the photographs (i.e., too unfocused).

**Fig 3:**
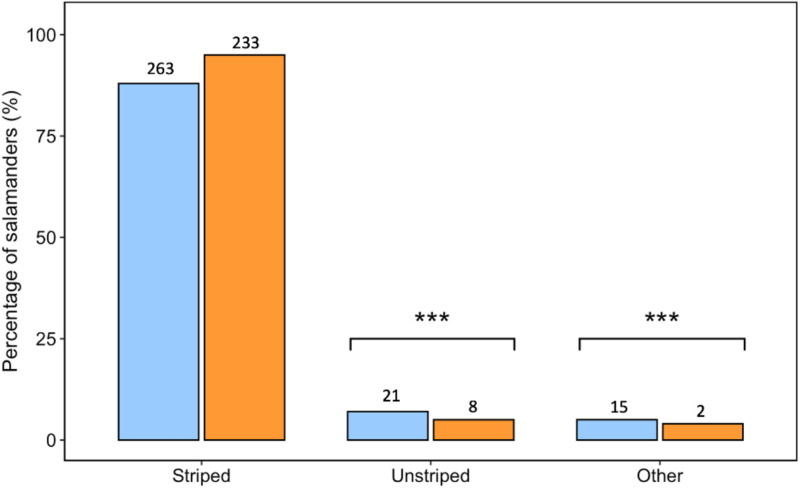
The percentage of Eastern Red-backed Salamanders (*Plethodon cinereus*) colour morphs reported on iNaturalist (blue bars) and observed during field surveys (orange bars). Data is presented by colour morph, which includes the striped or red-backed morph, the unstriped or lead-backed morph, and any other colour morphs observed (including erythristic, albino, amelanistic, or leucistic). In the graph, sample size is denoted above each column. Significance (*p* < 0.05) is denoted by asterisk’s (***).

In a comparison of the colour morphs observed during field surveys *versus* reported on iNaturalist, we found a significant relationship between the data collection method and colour morph frequencies (*χ*^*2*^_*2*_ = 11.92, *p* < 0.01) (Fig 3). Both methods were similar in how likely they would observe/report striped morphs (*χ*^*2*^_*2*_ = 1.81, *p* = 0.18), but community-scientists were more likely to report the less common colour morphs (unstriped: *χ*^*2*^_*2 *_= 5.83, *p* = 0.02 and other: *χ*^*2*^_*2 *_*= *9.94*, p* < 0.01).

### Social behaviour summary and comparison

We summarized how occurrences of adult (SVL > 32 mm) *P. cinereus* aggregations, including both mating and social aggregations, varied over time in New Brunswick across their active season (May to October). With our ecological survey data, we defined mating pairs as two adult salamanders of opposite sexes found within 30 cm of each other, whereas mating aggregations consist of more than two adult salamanders, all within 30 cm of one another [15]. To be classified as a mating aggregation, every individual had to meet the 30 cm distance requirement with all other individuals in the group. We combined data on mating pairs and mating aggregations to describe temporal differences among salamanders in these aggregations (Table 1). We also described seasonal trends in solitary individuals and social aggregations, of which the latter were when individuals were > 30 cm from one another but sharing the same shelter object.

**Table 1:** The aggregative behavior of *P. cinereus* across its active season (May to October 2022) encountered during our field surveys.

Month	Total individuals found	Mating aggregation (%)	Social aggregation (%)	Solitary (%)
May	28	21.4%	10.7%	67.9%
June	65	7.69%	9.23%	83.1%
July	33	12.1%	21.2%	66.7%
August	79	10.1%	31.6%	58.2%
September	31	6.45%	41.9%	51.6%
October	7	100%	0%	0%

Data includes the month the salamander was found, the total number of salamanders found that month, the percentage of salamanders that were found in mating aggregations (i.e., in a group of 2 or more within 30 cm of another adult *P. cinereus* of the opposite sex), social aggregations (i.e., under the same cover object but not part of a mating group), and solitary.

Social aggregations of *P. cinereus* in New Brunswick ranged in size from 2 to 10 individuals, with juveniles frequently co-occurring alongside adults. Smaller aggregations (2–3 individuals) typically consisted of one or two adults and juveniles, while larger aggregations (4–10 individuals) often included a mix of multiple adults and juveniles or neonates. Detailed information on aggregation composition is provided in the supplementary materials (Table S3 in S1 File).

**Fig 4:**
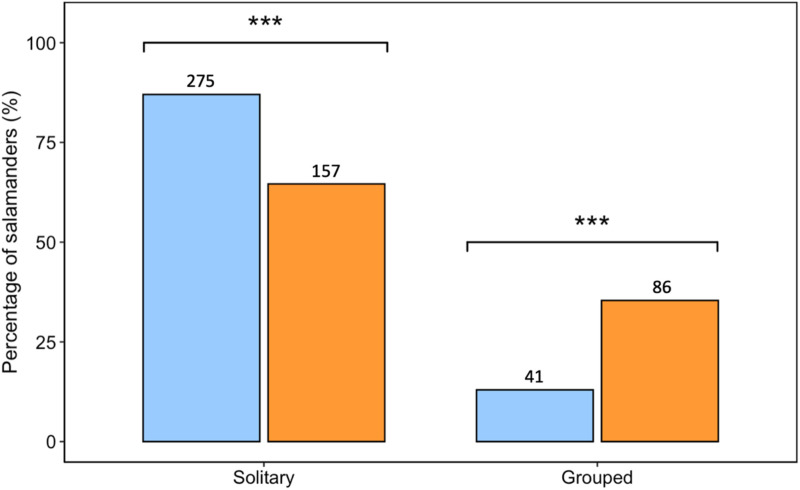
The percentage of Eastern Red-backed Salamanders (*Plethodon cinereus*) found aggregated under the same shelter object (grouped) *versus* found solitary under shelter reported on iNaturalist (blue bars) and during field surveys (orange bars). The sample size is denoted above each column. Significance (*p* < 0.05) is denoted by asterisk’s (***).

To investigate whether documentation of *P. cinereus* aggregative behaviour differed between field and iNaturalist datasets, we first ensured they were consistent. To do this, in our field dataset, we pooled the number of salamanders found in mating aggregations and pairs (*n* = 32) with the number found under the same shelter objects (i.e., social groups) (*n* = 54) to facilitate a comparison of this species’ aggregative behaviour across data sources. This was a step we took because one is unable to assess salamander sex and distance from one another in iNaturalist photographs. Based on this classification, in the field we observed a total of 86 *P. cinereus* in groups under the same shelter object with at least one other individual, and 157 *P. cinereus* alone (i.e., solitary) under cover. Within the iNaturalist dataset, we classified 41 salamanders as being a part of an aggregation either through photographs (*n* = 18) or through user comments (*n* = 23), and we classified 275 individuals as solitary. We then compared the aggregative tendency of *P. cinereus* documented in our field surveys versus reported on iNaturalist and we found a significant relationship between the method of data collection and *P. cinereus* aggregation status (*χ*^*2*^_*2*_ = 39.31, *p* < 0.001) (Fig 4). Community scientists were less likely to observe/report groups of *P. cinereus* and more likely to observe/report solitary individuals.

### Environmental factors and habitat selection

All data presented in this section were exclusively derived from our field study. Instead of statistical comparisons, we provide a summary of *P. cinereus* colour morph microclimate environmental preferences due to a small sample size of unstriped morphs. Qualitatively, the soil temperature and moisture, canopy cover, and cover object volume were similar between locations selected by each morph in our study (Table 2), yet this assessment is extremely limited due to the small sample size of unstriped morphs.

**Table 2:** A summary of environmental variables (average ± standard deviation) of adult unstriped and striped colour morphs of *P. cinereus* found during field surveys.

	Colour morph	
Environmental variable	Unstriped	Striped
Canopy cover (%)	57.83 ± 15.63 (*N*_*obs *_= 7)	65.32 ± 16.19 (*N*_*obs* _= 148)
Soil temperature (^o^C)	16.62 ± 2.50 (*N*_*obs* _= 6)	16.27 ± 3.52 (*N*_*obs *_= 151)
Soil moisture (%)	25.10 ± 10.73 (*N*_*obs* _= 6)	23.48 ± 17.54 (*N*_*obs* _= 149)
Cover object volume (m^3^)	406.51 ± 288.56 (*N*_*obs *_= 7)	313.10 ± 339.24 (*N*_*obs *_= 151)

Environmental variables were not statistically analyzed due to the limited sample of unstriped morphs. Environmental variables for juvenile *P. cinereus* (SVL < 32 mm) were not recorded.

We also examined whether environmental factors and cover object size differed between solitary and mating pairs/aggregations of salamanders. We did not find a relationship between salamander grouping tendency with mates and soil moisture (%), soil temperature (^o^C), cover object volume (m^3^), or canopy coverage (%) (Table 3).

**Table 3:** Outcomes of generalised linear mixed-effect models (GLMMs) testing if microclimate variables (i.e., soil moisture, canopy coverage, and soil temperature) and cover object volume differ between field locations where solitary and mating pairs/aggregations of Eastern Red-backed Salamanders (*Plethodon cinereus)* occur.

		Fixed effects				Random effect		
Environmental variable	Predictor	*β*	*SE*	*z*	*p*		*σ* ^*2*^	*SE*
**Soil moisture (%)**	Intercept	−0.096	2.591	−0.037	0.971	Study site	4.278	2.068
*N*_*obs*_* *= 143, *N*_*sites*_* *= 20	Soil moisture	0.026	0.014	1.916	0.055			
	Sex (male)	0.231	0.490	0.471	0.638			
	SVL	−0.070	0.065	−1.076	0.282			
**Soil temperature (** ^**o**^ **C)**	Intercept	1.860	2.959	8.302	0.530	Study site	2.964	1.722
*N*_*obs*_* *= 145, *N*_*sites*_* *= 20	Soil temperature	−0.162	0.102	−1.689	0.114			
	Sex (male)	0.342	0.481	0.742	0.477			
	SVL	−0.036	0.062	0.023	0.562			
**Cover object volume (m**^**3**^)	Intercept	0.459	2.802	0.164	0.870	Study site	3.181	1.783
*N*_*obs*_* *= 144, *N*_*sites*_* *= 20	Cover object volume	4.736	8.762	0.541	0.589			
	Sex (male)	0.025	0.532	0.0468	0.963			
	SVL	−0.074	0.071	−1.049	0.294			
**Canopy coverage (%)**	Intercept	−0.912	3.181	−0.287	0.774	Study site	3.42	1.848
*N*_*obs*_* *= 125, *N*_*sites*_* *= 19	Canopy cover	0.005	0.032	0.142	0.887			
	Sex (male)	0.331	0.482	0.687	0.492			
	SVL	−0.041	0.063	−0.647	0.518			

Reference levels for each categorical variable are shown in parentheses following variable names. For each model, we present coefficient estimates (*β*) and their corresponding standard error (*SE*), *z*-values (*z*), and *p*-values (*p*) for fixed effects, as well as variance (*σ*^*2*^) and *SE* of random effects. Model outputs are shown on the latent scale. *N*_*obs*_ refers to the number of observations where *N*_*sites*_ refers to the number of sites surveyed.

## Discussion

We documented the colour polymorphism and social behaviour of this species in notable spatial detail provincially within New Brunswick, as well as compared methods of data collection (e.g., field observations *versus* a community-science approach) using 243 field observations collected during this species’ 2022 active season and 316 community-science observations of this species from 2008–2022 posted on iNaturalist. We investigated whether preferences for micro-environmental factors, such as cover object size, soil moisture and temperature, and canopy cover, vary between color morphs and between aggregated versus solitary salamanders. However, in New Brunswick, we found no evidence of such preferences. Although we were unable to statistically compare whether preferences for micro-environmental factors differed between colour morphs, we do provide a summary of this data and found that environmental variables were similar between the striped and unstriped morph in our study. Our findings also indicate biases in iNaturalist data, with rare colour morphs being overrepresented and social groups underrepresented. Notably, two rare colour morphs – leucistic and amelanistic – were identified, expanding the known phenotypic diversity of this species in New Brunswick, Canada.

Rare colour morphs were commonly reported through iNaturalist, showing the platform’s capacity to record visually distinctive phenotypes. This included the leucistic morphs (observed by Bradley Doiron and Gregor Jongsma); one of which had been previously formally described in a natural history note [27]. Additionally, the iNaturalist dataset contained an amelanistic colour morph (observed by Damien Mullin), which to our knowledge, has yet to be formally documented in New Brunswick [8]. Although the most likely factor causing these rare phenotypes in *P. cinereus* are rare developmental anomalies (i.e., birth defects) the factors influencing their persistence remain largely unknown [8,16]. Thus, documenting their presence is a step towards understanding the underlying factors shaping their distribution and persistence. In animal populations, atypical colouration is tends to be most commonly found in young individuals because it can be maladaptive and decrease an individual’s fitness (e.g., survival decreased through higher predation, mating ability reduced due to non-ideal colouration) [8]. But, in *P. cinereus* these rare colour morphs are often documented as adults, and one reason why theses atypically coloured salamanders survive from the juvenile to adult lifestage is that their cryptic behaviour (i.e., spending a lot of their time under cover objects) affords camouflage against a variety of different backgrounds in their natural environment [8].

In addition to investigating the occurrence of *P. cinereus* colour morphs in New Brunswick, we also investigated whether an individual’s colouration affected the likelihood of finding and reporting these salamanders using different data collection methods. We found that both methods (e.g., field survey and community science) were similar in how likely they would observe/report the striped morph, but data collection using a community-science approach was more likely to observe/report less common and unique colour morphs (e.g., unstriped and others). These results are similar to a study that investigated differences between community-science and field data on the colour morph frequency of Eastern Gray Squirrels (*Sciurus carolinensis*) and found that iNaturalist was more likely to observe/report the novel white phenotype of Eastern Gray Squirrels (e.g., leucistic and albino squirrels) [23]. Thus, our results add to evidence that iNaturalist records of animal colouration are likely to be biased towards unique phenotypes and that the assumption that participants are likely to report observations of all phenotypes equally is unlikely true [23]. However, an alternative explanation to our study’s findings is that the broader geographic coverage of iNaturalist records in northern New Brunswick may have captured populations with higher frequencies of rare morphs that were not part of our field study. This said, there is not a known geographic cline in the occurrence of colour morphs in this province [8]. Overall, knowledge of potential biases emphasizes the need for cautious interpretation of iNaturalist data in research, which may be an important consideration when using this type of data in future colour science studies.

In addition to comparing methods of data collection for *P. cinereus* colour morphs, we also compared methods of data collection for this species’ social behaviour. iNaturalist has been previously used to study the nature of animal interactions, for example investigating human-wildlife interactions using bird sighting records [28], assessing predator-prey interactions through photographed injuries of Southern Alligator Lizards (*Elgaria multicarinata*) [29], and studying interactions between hummingbirds and pollinators using plant visitation records from both iNaturalist and eBird [30]. In this study, we compared the aggregative behaviour of *P. cinereus* observed during our field surveys, to the aggregative behaviour of *P. cinereus* recorded on iNaturalist. We found that a community science approach was less likely to report/observe aggregations of *P. cinereus* compared to an ecological survey approach. This underrepresentation of groups on iNaturalist may result from users prioritizing high-quality photographs, potentially leading to instances where participants relocate *P. cinereus* for better quality photos (e.g., holding the salamanders in their hand), thereby limiting our ability to accurately assess their aggregation status through the images. On the contrary, it is also possible that some of the grouped *P. cinereus* observed through iNaturalist photographs may have been placed alongside one another for a similar reason: to enhance the aesthetic appeal of the photo. This bias, alongside the bias we uncovered regarding users’ more frequent uploading of rare colour morphs to iNaturalist, seem to arise as users prioritize capturing visually appealing photographs, thus if users solely focused on documenting and identifying the species, such biases could be potentially minimized. Given these limitations, we do not recommend relying on iNaturalist data alone to study social behaviour of *P. cinereus* or similar species. Instead, iNaturalist should be used to gain broad-scale information about a species, such as documenting colour variation across geographic regions and increasing our understanding of the diversity of phenotypes that exist (rather than proportion of relative occurrence within populations).

Regardless of potential biases in community science datasets, there are still many advantages to this type of data that could not be possible with ecological data alone. Firstly, because community science-generated data covers a large geographic extent, the use of this data allowed evaluation of both the colouration and sociality of *P. cinereus* across a broader spatial scale. This is in contrast to our ecological dataset, which was more limited to the southern regions of New Brunswick. However, the reliability of community science data, especially regarding photo quality and behavioral assessments, remains a concern. For example, poor photo quality, repeated uploads by the same user, and uncertainty while assessing interactions in photos. Despite these challenges, our results suggest that community science data are valuable for detecting broad-scale patterns in colour morph frequencies, as long as biases, such as the overrepresentation of rare morphs, are taken into account. Efforts to address these biases are underway. For instance, methods developed to account for uneven sampling intensity, as described by Crossley et al. (2022), could enhance the reliability of community science data in future studies [31]. Despite its limitations, community science remains a powerful tool for science communication, expanding access to ecological information, and providing valuable novel insights into wildlife occurrences and biology.

We were not able to analyze environmental differences between striped and unstriped morphs in this study due to the small sample size of unstriped salamanders in our field dataset. Notably, both datasets (field and community science) contained low numbers of unstriped individuals compared to reports from other studies [15]. Given this small sample size, additional studies with larger numbers of unstriped salamanders are needed to determine whether environmental differences between morphs exist at the northern limits of *P. cinereus*’ range.

Studies on the social dynamics of the Eastern Red-backed Salamanders are extremely limited in Canada. The only work thus far has occurred in Ontario [20]. We provided a summary of the grouping behaviour of *P. cinereus* across its active season (May – October) in New Brunswick, marking the first investigation of social behaviour in this species within the province and at its northern Canadian range limit. Our data reveal seasonal variation in their social behavior with most mating groups forming near the fall. Specifically, we observed an increase in aggregated salamanders (including both mating groups and social groups) during late summer (August) and September, with a notable peak in October where all observed individuals were found in mating groups. This pattern likely reflects this species’ mating behavior, as previous research shows that *P. cinereus* breeding season is in the early spring or fall, during which males actively seek out females and aggregate with them [15].

We also tested whether environmental factors (soil moisture, soil temperature, and canopy cover) and cover object size differed between solitary and mating groups of *P. cinereus*. We found that environmental factors and cover object size did not significantly differ between solitary and mating groups of *P. cinereus.* This contrasts with findings from Quinn and Graves (1999), who reported that salamander aggregations were associated with higher soil moisture; however, their study included both mating and non-mating individuals, whereas we focused specifically on mating groups [24]. Our results suggest that, unlike general aggregative behavior, mating group formation in *P. cinereus* may not be strongly driven by microhabitat variation and may instead reflect behavioral or social cues independent of micro-environmental conditions.

This study is a first in New Brunswick Canada to include data from *P. cinereus* populations across 23 different forests and pair this with community-science data to increase our knowledge about this species’ natural history. Through this integrative approach, we compiled a comprehensive picture of this species’ natural history in New Brunswick, where information remains scarce, despite its large geographic range in this region. Importantly, our findings highlight the strengths of community-science data, such as its ability to capture broad-scale patterns and detect rare phenotypes across expansive geographic areas, while also underscoring its limitations, including overestimating the frequency of rare phenotypes and its unreliability for accurately assessing behavioral interactions in photographs. By identifying these strengths and weaknesses, we provide critical insights for optimizing the use of community-generated data in future studies of organismal biology.

## Materials and methods

### Study species

*Plethodon cinereus* is a lungless plethodontid salamander commonly found across eastern North America, specifically from southeastern Canada southward to northern North Carolina and from the Atlantic Coast northwestward to the northern Mississippi River [32]. In Canada, they occur in Ontario, east through Quebec and into the Maritime Provinces [8]. There is also an introduction of *P. cinereus*, outside of its native range, in Newfoundland, Canada [33]. This species is terrestrial at all life-stages and can be found on the forest floor where they forage for invertebrates within the leaf litter and often take refuge under cover objects like rotting logs and rocks [32].

### Study sites and field methods

We surveyed for Eastern Red-backed Salamanders in 23 New Brunswick forests from May to August 2022 and then resampled 7 of these sites from September to October 2022 to examine whether this species’ tendency to aggregate changes seasonally (Table S2 in S1 File). For each resampled site, we selected a new sampling location within the same general area but ensured it was at least 25 meters from the previous sampling location to minimize the likelihood of observing the same individuals or groups twice. Thus, as we minimised chances of pseudoreplication, data from all study sites were included in our analyses. Study sites consisted of protected areas, public walking trails and parks, and private properties. Sites were mostly mixed forests that contained both deciduous and coniferous trees (**n* *= 18), yet some were predominately deciduous forests (*n* = 5) (S2 Table in S1 File). Elevations of sites ranged from −56 m to 190 m, and soil type was either mineral or peat mix.

Initially at each site we moved through the forests haphazardly looking for an area with many shelter objects for salamanders. We then used a circular transect survey method with a radius of 25 m and walked from the center point of the circle along a series of straight lines while flipping cover objects until we covered the entire area (1962 m^2^). Whenever a salamander was found under a cover object, it was captured. We then measured and recorded salamander snout-vent length (SVL; mm) (i.e., the distance from the snout to the end of the cloacal opening), total length (mm) (i.e., the distance from the snout to the end of the tail), mass (g) using a MAXUS Elite digital pocket scale (capacity: 500 g), age (sexually mature individuals, or adults, have a SVL > 32 mm) [34], and then, for adult salamanders only, determined sex by candling (i.e., examination of the venter with a light source for the presence of testes in males and eggs in gravid females [35]). Salamanders were handled with nitrile gloves and measures to prevent disease spread were implemented during data collection [36].

If more than one salamander was under a cover object, we noted the total number of individuals (adults and juveniles) located under it, as a measure of aggregation tendency of salamanders at each site. We included juvenile salamanders when comparing the aggregation status between ecological and field data, because age (i.e., length of individuals) could not be assessed through iNaturalist photographs (Table S3 in S1 File). This kept both datasets consistent. Only mating pairs/groups were included when assessing whether environmental factors influenced their aggregation tendency (Table S3 in S1 File).

Lastly, for each cover object that adult salamanders were found under, we measured environmental factors associated with these sites. Once the cover object was flipped and the salamander(s) were removed, we measured the length, width, and height dimensions of the log or rock using a measuring tape. We also measured soil moisture (%) using a SM150 Soil Moisture Sensor (Delta-T Devices) and soil temperature (^o^C) using an infrared thermometer. Cover objects were placed back in their original location and canopy cover was measured 30 cm directly above using a spherical concave densiometer (43887 Model A, Universal Field Supplies). After data were collected on the salamanders and environmental factors, the salamanders were released back under cover and their shelter object was flagged to prevent resampling. This fieldwork was conducted under authorization from The Department of Natural Resources and Energy Development, Government of New Brunswick (scientific permit # SP22-002), and the Mount Allison University Animal Care Committee (protocol # 103181).

### Data extraction from iNaturalist

Data on occurrences of *P. cinereus* in New Brunswick were downloaded from iNaturalist on 23 September 2022. The dataset comprised 315 ‘research grade’ observations of Eastern Red-Backed Salamanders uploaded between 2008 and 2022. To be considered ‘research grade’, the observation must have a 2/3 consensus on the identification of the species, as well as include a photograph and location for the sighting. Each observation in our dataset included a digital photograph, user comments, the time, date, and location of the occurrence all submitted by a community member. This was then reviewed and verified regarding location of the observation, contributor identity, and the date and time the contributor uploaded the observation. One researcher (AM) then identified the colour polymorphism of the salamander in the uploaded photo. Low-quality photographs in which colour polymorphism could not be confidently determined (e.g., they were heavy shaded or zoomed-in) were not included (*n* = 17) in the dataset. The same researcher also assessed *P. cinereus* aggregation status by whether salamanders were within a group in the photograph or if grouping status was noted in the user comments (i.e., user states that multiple individuals were found under shelter together).

### Statistical analyses

All analyses were performed in R version 4.22 [37]. Data were explored before analyses to ensure it met model assumptions, such as no unexplainable outliers or collinearity between predictor variables [38]. For all statistical tests, *α* was set at 0.05. After tests were run, we ensured assumptions were met (e.g., normality of residuals for fixed and random effects, homogeneity of variance, overdispersion) by using the *‘check_model’* function from the R package *‘performance’* [39].

### Comparing ecological and community-science survey methods

We summarized the percentage of striped, unstriped, and other (i.e., any other than striped or unstriped) *P. cinereus* colour morphs separately for the field and iNaturalist data. We then statistically compared the percentage estimates of *P. cinereus* colour morphs generated from field and community-science data using a *χ*^*2*^-test [40]. This test was performed using the R function *‘chisq.test’* from the *‘*stats*’* R package [38] and results from the *χ*^*2*^-test were visualised using the R function *‘ggbarstats’* from the R package *‘ggstatsplot’* [40].

To compare the aggregative tendency of *P. cinereus* between methodologies, we summarized the percentage of salamanders that were found solitary and that were found grouped separately for the field and iNaturalist dataset. In the field dataset, we pooled mating pairs/groups (i.e., two or more adult salamanders of opposite sexes found within 30 cm of each other) and social groups (i.e., individuals > 30 cm from one another but sharing the same shelter object). For the iNaturalist dataset, a group was classified as 2 or more individuals of *P. cinereus* in the same photograph, or found under the same shelter object as indicated by the user’s comments. We then statistically compared the percentage estimates of *P. cinereus* found solitary versus found in groups generated from the field and community-science dataset with a *χ*^*2*^-test using the same process in R as described above [37,40,41]

### Environmental factors and habitat selection

We examined habitat selection of grouped (within mating pairs/groups only) and solitary *P. cinereus* in New Brunswick. To accomplish this, we ran four GLMMs using the function *‘glmer’* from the *‘lmerTest’* R package [42] to analyse whether the response variable of grouped or not (binomial variable where within a group = 1 and solitary = 0) was impacted by soil moisture (%), soil temperature (^o^C), cover object volume (m^3^) or canopy cover (%). In our analyses, canopy cover and soil moisture were converted to proportions [43]. In addition to each of the environmental variable listed above, the four binomial GLMMs also contained the fixed effects of sex (categorial variable: female and male) and SVL (mm; continuous variable), as well as the random effect of study site.

## Supporting information

S1 File Supplementary Materials_McCormick and Riley 2025. This is one file containing Table S1, Table S2, and Table S3 that details additional information about colour morphs of *Plethodon cinereus*, information about study site locations, and the composition of non-mating groups observed in this study.
(PDF)
