# Supplementary material for: Integrating ecological and community science data to understand patterns of colour polymorphism and social behaviour at the northern range limit of a plethodontid salamander
Source: PLoS One. 2025 Sep 23;20(9):e0332501. doi: 10.1371/journal.pone.0332501 (PMC12456789; doi:10.1371/journal.pone.0332501)
Supplement: S1 File — This is one file containing Table S1, Table S2, and Table S3 that details additional information about colour morphs of Plethodon cinereus, information about study site locations, and the composition of non-mating groups observed in this study. (PDF) [file pone.0332501.s001.pdf]

Integrating ecological and community science data to understand patterns of colour polymorphism and social behaviour at the northern range limit of a plethodontid salamander

Alexia McCormick and Julia L. Riley

Supplementary Materials

The supplementary materials contain additional information about the distribution of *P. cinereus* colour morphs across their geographic range, the location where field surveys were conducted in this study, as well as composition of non-mating groups that were observed during our field surveys.

Tables

Table S1. The percentage of Eastern Red-backed Salamander (*Plethodon cinereus*) colour morphs reported across their geographic range. Data was acquired from a non-systematic search of literature. Color morphs include, striped, unstriped and other, and *n* indicates the sample size of individuals surveyed.

		% of *P. cinereus* colour morphs				
**Location**	**Latitude, Longitude**	**Striped**	**Unstriped**	**Other**	***n***	**Citation**
Odell Park, Fredericton, NB, CAN	45.572260°N, -66.395448°W	83	7	10	103	5
Point Pleasant Park, NS, CAN	44.626067°N, -63.573166°W	61	39	0	48	8
Point Pleasant Park, NS, CAN	44.626067°N, -63.573166°W	60	40	0	20	8
Mount Mégantic, Montreal, QC, CAN	45.450000°N, -71.150000°W	99	1	0	101	7
Mount Royal, Montreal, QC, CAN	45.500000°N, -73.583333°W	97	3	0	120	7
Central Ontario, CAN	---	78	22	0	2144	6
Ringwood, NY, USA	---	100	0	0	161	4
Cuyahoga Valley National Park, OH, USA	41.228931°N, -81.518511°W	78	22	0	224	1
Cuyahoga Valley, National Park, OH, USA	41.229617°N, -81.518825°W	77	23	0	188	2
Central Pennsylvania, USA	---	68	32	0	51	3
New Haven, CT, USA	41.317222°N, -72.751944°W	67	33	0	>100	9
Bristol County, MA, USA	41.952222°N, -71.168611°W	88	9	2	>100	9

[1]Acord et al., 2013; [2]Anthony et al., 2008; [3]Brown, 1965; [4]Davis and Milanovich, 2020; [5]Jongsma, 2012; [6]Morneault et al., 2004 ; [7]Noël et al., 2007; [8]Russell et al., 2011 ; [9]Lotter and Scott, 1977

Table S2. Study sites where surveys were completed in New Brunswick. The type of forest (deciduous, coniferous, or mixed), number of surveys completed at each site, the number of salamanders found at each site (*n*), and general location of the site are also detailed. If multiple surveys were completed at a site (including re-sampling) then they began at different center points that were at least 50 m away from each other.

Study Site	Forest Type	No. Surveys	*n*	Latitude (°N)	Longitude (°W)
Atlantic Wildlife Institute, Cookville	Mixed	2	9	46.019056	-64.312141
Beach Hill Park, Sackville	Mixed	2	33	45.947305	-64.401771
Claude D. Taylor School, Riverview	Mixed	2	12	46.050628	-64.818338
Irishtown Nature Park, Riverview	Mixed	3	24	46.142254	-64.772009
Lower Walker Rd. Trails, Sackville	Mixed	4	36	45.931778	-64.406939
Mapleton Park, Moncton	Mixed	1	6	46.125254	-64.834051
Millcreek Nature Park, Riverview	Mixed	2	4	46.054798	-64.766586
NCC Reserve # 22, Cookville	Deciduous	2	6	46.110723	-64.572065
Odell Park, Fredericton	Deciduous	1	43	45.949589	-66.678509
Private Property 1, Sackville	Deciduous	1	10	45.962104	-64.408868
Private Property 2, Sackville	Deciduous	1	7	45.962132	-64.408856
Private Property 3, Sackville	Mixed	2	4	45.975515	-64.470876
Private Property, Shediac	Mixed	1	6	46.112490	-64.583635
Private Property 1, Summerville	Mixed	2	12	45.352821	-66.089468
Private Property 2, Summerville	Mixed	2	12	45.353310	-66.088226
Private Property, Lakeside	Mixed	2	19	45.502690	-65.790055

Table S3. Composition of non-mating aggregations (salamanders > 30 cm from one another but sharing the same shelter object) and mating aggregations (two or more adult salamanders of opposite sexes found within 30 cm of each other) of Eastern Red-backed Salamanders (*Plethodon cinereus*) observed in New Brunswick. The table presents group sizes and the corresponding distribution of adult and juvenile individuals across various group compositions.

**Non-mating aggregations**	
**Aggregation size**	**Aggregation composition details**
I individual*	3 separate groups (1 juvenile, 1 male, and 1 female)
2 individuals	9 groups: 1 adult (observed 7 times with a female and 2 times with a male) and 1 juvenile<br>3 groups: 2 juveniles<br>3 groups: 2 adults
3 individuals	1 group: 1 adult female and 2 juveniles
4 individuals	1 group: 1 adult female and 3 juveniles<br>1 group: 1 adult male and 3 juveniles
10 individuals	1 group: 1 adult male, 2 unsexed adults, and 7 neonates
**Mating aggregations**	
**Aggregation size**	**Aggregation composition details**
2 individuals	13 groups: all adult male/female pairs
3 individuals	2 groups: 2 adult males and 1 adult female

* These three single-individual non-mating groups were each found under the same shelter object as a mating pair but were not classified as part of the mating group. Under the group definition criteria (i.e., individuals sharing a shelter object), these individuals were recorded as separate non-mating groups.

References

Acord MA, Anthony CD, Hickerson C. Assortative Mating in a Polymorphic Salamander. Copeia. 2013; 2013(4): 676-683. doi: 10.1643/CE-13-003

Anthony CD, Venesky MD, Hickerson C.A.M. Ecological Separation in a Polymorphic Terrestrial Salamander. J. Anim. Ecol. 2008; 77(4): 646-653. doi: 10.1111/j.1365-2656.2008.01398

Brown JL. Stability of Colour Phase Ratio in Populations of *Plethodon cinereus*. Copeia. 1965; 1965(1): 95-98. doi:10.2307/1441246.

Davis AK, Milanovich JR. Lead-phase and Red-stripe Colour Morphs of Red-backed Salamanders *Plethodon cinereus* differ in hematological stress indices: A Consequence of Differential Predation Pressure? Curr. Zool. 2010; 56(2): 238-243. doi:10.1093/czoolo/56.2.238

Jongsma G. Co-occurrence of Three Colour Morphs of *Plethodon cinereus* (Eastern Red-backed Salamander) in Odell Park, Fredericton, New Brunswick, Canada. Herpetol. Rev. 2012; 43(2): 318.

Morneault AE, Naylor BJ, Schaeffer LS, Othmer DC. The Effect of Shelterwood Harvesting and Site Preparation on Eastern Red-backed Salamanders in White Pine Stands. Ecol. Manag. 2004; 199(1): 1-10. doi: 10.1016/j.foreco.2004.03.043.

Noël S, Ouellet M, Galois P, Lapointe FJ. Impact of Urban Fragmentation on the Genetic Structure of the Eastern Red-backed Salamander. Conserv. Genet. 2007; 8(3): 599-606. doi: 10.1007/s10592-006-9202-1.

Lotter F, Scott NJ. Correlation between Climate and Distribution of the Colour Morphs of the Salamander *Plethodon cinereus*. Copeia. 1977; 1977(4): 681-690. doi:10.2307/1443166.

Russell RW, Beslin W, Hudak M, Ogunbiyi A, Withrow A, Gilhen J. A Second Amelanistic Eastern Red-backed Salamander, *Plethodon cinereus*, from Nova Scotia, Canada. Can. Field-Nat. 2011; 125(4): 359-372. doi: 10.22621/cfn.v125i4.1265.
